# Socioeconomic and Spatial Determinants of Dog Abandonment and Adoption in the Republic of Korea (2021–2023)

**DOI:** 10.3390/ani15111613

**Published:** 2025-05-30

**Authors:** HyungChul Rah

**Affiliations:** Department of Companion Animal and Plant Sciences, College of Future Convergence, Jeonju University, Jeonju 55069, Republic of Korea; hrah.joshua@jj.ac.kr

**Keywords:** dog abandonment, adoption, socioeconomic factors, veterinary costs, spatial dependence

## Abstract

This study examined the socioeconomic and veterinary cost factors influencing dog abandonment and adoption outcomes across 162 regions in the Republic of Korea from 2021 to 2023. Unlike prior research, this study distinguished between intentionally abandoned dogs and those lost and subsequently returned to their owners, normalizing abandonment data by population. Using publicly available regional data and spatial regression models, we found that the number of people receiving unemployment benefits was consistently and negatively associated with the number of dog abandonments per 100,000 residents, which was normalized by total population to avoid potential errors. Rabies vaccination costs were also negatively associated with abandonment. In contrast, comprehensive income tax amounts—a proxy for regional wealth—were positively correlated with the percentage of dog abandonments reported in 2021 and 2023. Spatial Lag Models accounted for over 50% of the variance in the number of dog abandonments, confirming spatial dependence and highlighting the importance of geographically targeted animal welfare interventions. However, spatial patterns in adoption were less consistent. These findings highlight the importance of incorporating economic and spatial considerations into the design of public policies and shelter strategies to mitigate dog abandonment and enhance adoption outcomes.

## Simple Summary

This study investigated the reasons behind dog abandonment and the factors influencing their adoption chances in the Republic of Korea. By using data from 162 regions between 2021 and 2023, this research focused only on dogs that were truly abandoned, excluding those that were simply lost and later returned to their owners. By combining regional statistics with veterinary service costs, this study found that areas with higher rates of unemployment benefits tended to have lower numbers of dog abandonment per 100,000 residents. This surprising result was revealed after the number of dog abandonments was normalized by the total population to avoid potential errors. This study also found that a higher percentage of abandoned dogs were adopted in wealthier regions, measured by comprehensive income tax data, especially in 2021 and 2023. Notably, this study used spatial analysis to identify geographical patterns, revealing that dog abandonment tends to cluster in specific areas. These insights underscore the necessity for region-specific policies, including accessible veterinary care and public support for companion animal guardians, to mitigate abandonment and enhance adoption outcomes.

## 1. Introduction

In our previous study, we demonstrated that both socioeconomic factors (e.g., tax burden and unemployment) and veterinary costs (e.g., X-ray and vaccination fees) were significantly associated with companion animal relinquishment in the Republic of Korea (ROK) [1]. However, that analysis grouped dogs and cats and did not distinguish between intentionally abandoned animals and those that were unintentionally lost and later returned to their owners. Such limitations may have introduced biases in understanding species-specific patterns of abandonment and their underlying causes.

The present study addresses these gaps by focusing solely on abandoned dogs, excluding lost animals, and analyzing abandonment and adoption outcomes separately across three years (2021–2023). We also incorporate spatial regression models to explore geographical dependencies in dog abandonment and survival rates, which are often overlooked in conventional regression analyses. This study specifically investigated the relationship between socioeconomic indicators and regional veterinary expenditures and two key outcomes: the rate of dog abandonments per 100,000 residents and the proportion of abandoned dogs subsequently adopted. By applying spatial analytical techniques and conducting year-over-year validation, this study aimed to provide a more accurate and geographically nuanced understanding of the socioeconomic drivers of dog abandonment and survival in the ROK [2].

## 2. Materials and Methods

Public data were collected to identify the factors contributing to the abandonment of dogs and the adoption of abandoned dogs in the ROK. For dogs, the number of dog abandonments and the percentage of dog abandonments adopted were selected as dependent variables because the average percentage of dog abandonments adopted from 2021 to 2023 was 44.6%, 38.9%, and 38.3%, respectively (see Table S1). For dog abandonment, one of the dependent variables, the number of abandonments by region, was calculated by subtracting the number of dogs returned to their owners from the total number rescued by public animal shelters. This figure was then standardized per 100,000 residents to yield the number of dog abandonments per 100,000 population, thereby minimizing potential bias due to population size or density, which may co-vary with several socioeconomic indicators. The percentage of dog abandonments adopted as another dependent variable was counted by dividing the number of dog abandonments adopted by the total number of dog abandonments.

The number of companion animal abandonments in 250 local cities, counties, and district governments was sourced from the 2021, 2022, and 2023 reports on animal abandonment by the Korean Animal Welfare Association (KAWA) [3,4,5]. Independent variables included veterinary costs by region and socioeconomic indicators by region (e.g., the number of people receiving unemployment benefits, comprehensive income tax amounts, and local income per region). The veterinary cost data for dogs from 4159 veterinary clinics and hospitals that were surveyed (online and on-site) and reported by region by the Ministry of Agriculture, Food, and Rural Affairs (MAFRA), the ROK, in 2024 (data available at https://www.animalclinicfee.or.kr/, accessed on 15 January 2025) include the following: initial examination fee amounts; re-visit examination fee amounts; consultation fee amounts; hospitalization fee amounts (for small dogs); DHPPL vaccination amounts; rabies vaccination amounts; kennel cough vaccination amounts (Bordetella vaccination); canine influenza vaccination amounts; complete blood count and reading fee amounts; and X-ray and reading fee amounts.

The public data on the number of people receiving unemployment benefits and comprehensive income tax amounts were collected as previously described [1]. The regional income per region from 2021 to 2023 was collected from the Korean Statistical Information Service. Data from 162 local governments were used for the dog abandonment among 250 local city, county, and district governments (see Table 1 and Table S1). The 2023 Korean map shapefile was obtained from the Geoservice database (data available at http://www.gisdeveloper.co.kr/?p=2332, accessed on 16 April 2025).

The total number of animals abandoned in a region was recoded as the number of dog abandonments per 100,000 residents in each region, since total population and population density would likely co-vary with many of the socioeconomic measures as well as access to and cost of veterinary care, and it could introduce a potential error to this study. Independent variables related to the number of dog abandonments per 100,000 residents and the percentage of dog abandonments that were adopted were estimated using multiple regression models. By using the veterinary cost data and the socioeconomic indicators in 2021 by region as independent variables, least squares multiple regression analyses were conducted to estimate the number of dog abandonments per 100,000 residents and the percentage of dog abandonments adopted in 2021 by region as dependent variables, using the IBM^®^ SPSS^®^ Software version 29.000 (IBM Corp. Released 2023, IBM SPSS Statistics for Windows, Version 29.0. Armonk, NY, USA: IBM Corp.). The same analyses were conducted to verify the findings using data from 2022 to 2023.

Spatial autocorrelation was used to investigate the presence of spatial clusters in the number of dog abandonments per 100,000 residents and the percentage of dog abandonments. Spatial regression models were applied using Ordinary Least Square (OLS) estimation to examine the correlation between the predictors and dependent variables, including the number of dog abandonments and the percentage of dog abandonments adopted, with the Geoda software version 1.22 [6].

## 3. Results

Section 3.1 presents the multiple regression models for the number of dog abandonments per 100,000 residents in 2021, 2022, and 2023. Subsequently, Section 3.2 presents the percentage of dog abandonments that occurred during the same period. Section 3.3 presents spatial regression models to account for the number of dog abandonments per 100,000 residents and the percentage of dog abandonments adopted from 2021 to 2023.

### 3.1. Regression Models for the Number of Dog Abandonments per 100,000 Residents from 2021 to 2023

Three socioeconomic status predictors and ten veterinary cost predictors, listed in Table 2, were used to develop a regression model that accounts for the number of dog abandonments per 100,000 residents in 2021. A multiple regression model with all thirteen predictors yielded an adjusted coefficient of determination (R^2^-adjusted) of 0.357 in explaining the number of dog abandonments per 100,000 residents in 2021, with a statistically significant *p*-value of less than 0.001. Based on the enter method with predictors with statistical significance, another multiple regression model with four predictors, including one socioeconomic status predictor and three veterinary cost predictors, presented a slightly decreased adjusted coefficient of determination of 0.348 in explaining the number of dog abandonments per 100,000 residents in 2021, with a significant *p*-value < 0.001. These four predictors included the number of people receiving unemployment benefits as a socioeconomic status predictor, as well as DHPPL vaccination amounts for dogs, rabies vaccination amounts for dogs, and canine influenza vaccination amounts, all of which served as veterinary cost predictors, with standardized coefficients of −0.343, 0.366, 0.530, and −0.192, respectively.

The findings on the number of dog abandonments per 100,000 residents in 2021 were to be verified by a multiple regression model using the 2022 data and the same enter approach. Thirteen independent predictors, including three socioeconomic status predictors and ten veterinary cost predictors (Table 3), were used to develop a regression model that accounts for the number of dog abandonments per 100,000 residents in 2022. A multiple regression model with thirteen predictors showed an adjusted coefficient of determination of 0.343 and statistical significance in explaining the number of dog abandonments per 100,000 residents in 2022, with a *p*-value of less than 0.001. It was followed by another regression model with two statistically significant predictors. It presented a slightly decreased adjusted coefficient of determination of 0.323, with significance in explaining the number of dog abandonments per 100,000 residents in 2022, as indicated by a *p*-value of less than 0.001. These two predictors included the number of people receiving unemployment benefits and the amount of rabies vaccinations for dogs, with standardized coefficients of −0.311 and −0.387, respectively.

The findings on the number of dog abandonments per 100,000 residents in 2021 and 2022 were to be verified by a multiple regression model using the 2023 data and the same enter approach. The thirteen independent predictors in Table 4 were used to develop a regression model that accounts for the number of dog abandonments per 100,000 residents in 2023. A multiple regression model with thirteen predictors showed an adjusted coefficient of determination of 0.345 and statistical significance in explaining the number of dog abandonments per 100,000 residents in 2023, with a *p*-value of less than 0.001. Another regression model was used, which included two statistically significant predictors. It presented a slightly decreased adjusted coefficient of determination of 0.321, with significance in explaining the number of dog abandonments per 100,000 residents in 2023, as indicated by a *p*-value of less than 0.001. These two predictors were the number of people receiving unemployment benefits and the amount of rabies vaccinations for dogs, with standardized coefficients of −0.293 and −0.403, respectively. Both were significant predictors in 2022, with standardized coefficients of −0.311 and −0.387, and in 2021, with standardized coefficients of −0.343 and −0.530.

### 3.2. Regression Models for the Percentage of Dog Abandonments Adopted from 2021 to 2023

The thirteen predictors in Table 5 were used to develop a regression model that accounts for the percentage of dog abandonments reported in 2021. A multiple regression model with all thirteen predictors yielded an adjusted coefficient of determination of 0.156 and statistical significance in explaining the number of dog abandonments in 2021, with a *p*-value of less than 0.001. Another multiple regression model, which included comprehensive income tax amounts as a socioeconomic status predictor, presented a slightly decreased adjusted coefficient of determination of 0.112 and statistical significance in explaining the number of dog abandonments in 2021, with a *p*-value of less than 0.001. The comprehensive income tax amounts were a statistically significant indicator with standardized coefficients of 0.343.

The thirteen independent predictors listed in Table 6 were used to verify the 2021 findings using the 2022 data on dog abandonments. A multiple regression model with thirteen predictors yielded an adjusted coefficient of determination of 0.064 and a *p*-value of 0.041, indicating significance in explaining the percentage of dog abandonments in 2022. Because there was no predictor with statistical significance, no further regression modeling was pursued.

The thirteen independent predictors listed in Table 7 were used to verify the findings of 2021 and 2022 by using the 2023 data on dog abandonments. A multiple regression model with the thirteen predictors showed an adjusted coefficient of determination of 0.079 and statistical significance in explaining the percentage of dog abandonments in 2023, with a *p*-value of 0.019. Another multiple regression model, which included comprehensive income tax as a predictor of socioeconomic status, was statistically significant in explaining the number of dog abandonments in 2023 (*p* = 0.007). However, it yielded a lower adjusted coefficient of determination (R^2^-adjusted = 0.039). Comprehensive income tax amounts were a statistically significant predictor of the percentage of dog abandonments in both 2021 and 2023, with standardized coefficients of 0.343 and 0.211, respectively.

### 3.3. Spatial Regression Models to Account for the Number of Dog Abandonments per 100,000 Residents and the Percentage of Dog Abandonments Adopted from 2021 to 2023

To measure the geospatial autocorrelation and clusters of the dependent variables, which include the number of dog abandonments per 100,000 residents and the percentage of dog abandonments adopted from 2021 to 2023, OLS estimation and spatial regression models were applied to examine the correlation between the predictors and dependent variables.

For the number of dog abandonments per 100,000 residents in 2021, both the Lagrange Multiplier (lag) and Lagrange Multiplier (error) test statistics were statistically significant, indicating the presence of spatial dependence (see Table S2). Additionally, the Robust LM (lag) and Robust LM (error) statistics were also substantial. Based on the spatial regression model decision rule [7], the SLM was selected because the order of magnitude of the LM (lag) was more significant than the Lagrange Multiplier (error). The Likelihood Ratio tests for both spatial models are substantial, indicating that each spatial model provides improvement over the OLS model. The SLM fits better than the SEM because the order of magnitude of the SLM is more significant than that of the SEM.

After comparing the results of the regression diagnostics determination coefficient (R^2^), Log-likelihood, Akaike info criterion (AIC), and Schwarz criterion (SC), the SLM was found to improve with the highest R^2^ of 0.543, indicating that the SLM explained 54.3% of the variance in the number of dog abandonments per 100,000 residents in 2021. In the SLM for the number of dog abandonments per 100,000 residents in 2021, the number of people receiving unemployment benefits, a socioeconomic status predictor, and rabies vaccination amounts, a veterinary cost predictor, were statistically significant and negatively associated with the number of dog abandonments per 100,000 residents (Table 8). In contrast, veterinary cost predictors, including consultation fee amounts, hospitalization fee amounts for small dogs, DHPPL vaccination amounts, canine influenza vaccination amounts, complete blood count and reading fee amounts, and X-ray and reading fee amounts, were significant and positively associated with the number of dog abandonments per 100,000 residents in 2021.

Regarding the number of dog abandonments in 2022, the Lagrange Multiplier (lag) and Lagrange Multiplier (error) test statistics were statistically significant, indicating the presence of spatial dependence (see Table S3). The Likelihood Ratio tests for both spatial models are substantial, and the SLM fits better than the SEM between the two spatial models, improving over the OLS model because the order of magnitude of the SLM is more significant than that of the SEM.

Based on the results of the regression diagnostics R^2^, Log-likelihood, AIC, and SC, the SLM was found to improve with the highest R^2^ of 0.505, indicating that the SLM explained 50.5% of the variance in the number of dog abandonments per 100,000 residents in 2022 compared to the other two models. In the SLM for the number of dog abandonments per 100,000 residents in 2022, the number of people receiving unemployment benefits, a socioeconomic status predictor, and re-visit examination fee amounts for dogs and rabies vaccination amounts, two veterinary cost predictors, were statistically significant and negatively associated with the number of dog abandonments per 100,000 residents (Table 8). However, veterinary cost predictors, including consultation fee amounts, hospitalization fee amounts for small dogs, DHPPL vaccination amounts, and X-ray and reading fee amounts, were significant and positively associated with the number of dog abandonments per 100,000 residents in 2022.

For the number of dog abandonments in 2023, both the Lagrange Multiplier (lag) and Lagrange Multiplier (error) test statistics were statistically significant, indicating the presence of spatial dependence (see Table S4). The Likelihood Ratio tests for both spatial models are substantial, and the SLM fits better than the SEM due to the order of magnitude difference between the two models.

Comparing the results of the regression diagnostics (R2, Log-likelihood, AIC, and SC), the SLM was found to improve, with the highest R^2^ of 0.506, indicating that the SLM explained 50.6% of the variance in the number of dog abandonments per 100,000 residents in 2023, compared to the other two models. In the SLM for the number of dog abandonments per 100,000 residents in 2023, the number of people receiving unemployment benefits, a socioeconomic status predictor, and re-visit examination fee amounts for dogs and rabies vaccination amounts, two veterinary cost predictors, were statistically significant and negatively associated with the number of dog abandonments per 100,000 residents (Table 8). In contrast, veterinary cost predictors, including consultation fee amounts, DHPPL vaccination amounts, and X-ray and reading fee amounts, were significant and positively associated with the number of dog abandonments per 100,000 residents in 2023.

Figure S1a–c shows the spatial distribution of the number of dog abandonments per 100,000 residents from 2021 to 2023. The spatial distribution pattern of the residual plot showing the difference between the predicted and observed values of the number of dog abandonments per 100,000 residents estimated by the Spatial Lag Model depicts that the spatial clusters of the number of dog abandonments per 100,000 residents are in regions with dark purple.

For the percentage of dog abandonments adopted in 2021 and 2022, none of the diagnostic tests for spatial dependence were statistically significant, indicating the absence of spatial dependence (Table 9). The Likelihood Ratio tests for either spatial model are not significant, suggesting that neither spatial model provides an improvement over the OLS model. In the 2021 dog abandonment data, comprehensive income tax amounts, a socioeconomic status predictor, and consultation fee amounts (in thousands of KRW), a veterinary cost predictor, were statistically significant and positively associated with the percentage of dog abandonments adopted (Table 9). However, in the 2022 data on dog abandonments, no independent predictor was statistically significant for the percentage of dog abandonments (Table 9).

For the percentage of dog abandonments adopted in 2023, Lagrange Multiplier (error) test statistics alone were statistically significant among the diagnostics for spatial dependence, indicating the presence of spatial dependence (Table 9). The Likelihood Ratio test for the Spatial Error Model alone is significant, indicating that the SEM provides improvement over the OLS model (see Table S5). In the SEM for the percentage of dog abandonments adopted in 2023, comprehensive income tax amounts, a socioeconomic status predictor, were statistically significant and positively associated with the percentage of dog abandonments adopted. However, consultation fee amounts (in thousands of KRW) and a veterinary cost predictor were not significant (see Table S5).

Figure S2a–c shows the spatial distribution of the percentage of dog abandonments adopted from 2021 to 2023. The more clustered regions were depicted by the SEM for the percentage of dog abandonments adopted in 2023 than by the OLS models for the percentage of dog abandonments adopted in 2021 and 2022.

## 4. Discussion

Our previous report presented that socioeconomic status and veterinary cost factors contribute to the increase in companion animal relinquishments in the Republic of Korea [1]. However, there were several limitations in the previous report, such as the number of animal relinquishments of dogs and cats being grouped into a single group. Furthermore, intentionally abandoned animals and unintentionally lost animals that were returned to the owners were counted as the number of animal relinquishments. Because the pattern of companion animal abandonment and the survivals of abandoned dogs and cats may differ [8,9,10], separate analyses for abandoned dogs and cats would provide a better understanding and lead us to a more appropriate approach to address the abandonments of dogs and cats. This study aimed to identify factors influencing the abandonment and survival of dogs in the Republic of Korea, with particular emphasis on socioeconomic variables, veterinary costs, and spatial dependencies. Our findings provide new insights that build upon our prior work by differentiating between species, isolating cases of abandonment from those of lost animals, and integrating geospatial analysis.

One of the key findings in this study is the consistent negative association between the number of people receiving unemployment benefits and the number of dog abandonments per 100,000 residents across all three years studied. This result suggests that when the number of dog abandonments was standardized by population size, areas with higher numbers of unemployment benefit recipients may have a lower number of dog abandonments per resident.

Additionally, rabies vaccination fees were negatively associated with dog abandonments during all three years, while DHPPL vaccination fees showed positive associations and canine influenza vaccination fees showed negative associations. These results suggest that the cost of essential preventive care may act as both a deterrent to utilization and an indicator of broader service access to veterinary services or owner engagement with animal healthcare. Rabies vaccination costs, often publicly funded, indicate the strength of public health infrastructure and the availability of free or subsidized preventive care. The availability of these subsidized vaccines is crucial, particularly as rabies is a legally mandated vaccination in many jurisdictions and is frequently supported through community-based campaigns. As highlighted in a Latin American study, the persistent classification of rabies as a public health threat underscores the need for sustained vaccination and prevention efforts [11]. Higher clinic-based rabies vaccine prices reflect reduced availability of publicly funded services. This may drive greater utilization of free community clinics and foster more regular engagement between dog guardians and public health systems. Such engagement could foster stronger human–animal bonds and improve retention, which emphasizes how structural access to veterinary care influences guardian behavior and animal welfare [12].

In contrast, DHPPL and canine influenza vaccines are often not subsidized, and their market-based pricing may reflect more elective or discretionary veterinary care. In lower-resource settings, increasing costs for non-mandatory vaccines may signal wider financial burdens, contributing to animal abandonment, particularly when combined with other socioeconomic challenges. This interpretation aligns with findings from studies in the United States and Brazil, demonstrating that actual and perceived veterinary costs can act as barriers to ownership of companion animals, especially in economically disadvantaged households [13,14].

Regarding the percentage of dog abandonments adopted, comprehensive income tax amount—a proxy for overall wealth—was the only variable showing positive association with the percentage of dog abandonments adopted consistently in 2021 and 2023. The consistently positive association between comprehensive income tax amounts and the percentage of adopted dogs suggests that communities with greater resources are more likely to adopt animals from shelters, possibly due to better facilities, community awareness, or education initiatives.

A significant contribution of this study is the identification of spatial dependence in dog abandonments across all three years. The Spatial Lag Model explained over 50% of the variance in abandonment cases during the study period, underscoring the importance of including geospatial effects in abandonment research. Regions with clusters of higher dog abandonment numbers could be prioritized for targeted intervention strategies, such as mobile spay/neuter clinics, low-cost vaccination programs, or local campaigns to promote responsible pet ownership. On the other hand, adoption rates exhibited no spatial dependence in 2021 and 2022; however, mild spatial clustering emerged in 2023, indicating that regional disparities in adoption infrastructure or public outreach may be growing and warrant further attention.

The results for the number of dog abandonments per 100,000 residents were opposite to those from two Korean studies, particularly the association between the number of people receiving unemployment benefits and the number of dog abandonments [1,10]. Previous studies analyzed the number of animal abandonments without adjusting for population size. In contrast, the present study normalized the number of dog abandonments by regional total population, which may account for differences in interpretation when examined alongside socioeconomic indicators. Although the data were not presented in this paper, our preliminary analysis showed that when the number of dog abandonments was used without recoding, a positive correlation was observed with the number of people receiving unemployment benefits, consistent with the prior Korean studies. However, the correlation became negative when dog abandonments were converted per 100,000 residents, as in the current analysis. This suggests that population size is a critical confounding factor in interpreting the relationship between unemployment and dog abandonments and that standardization by population may yield a more accurate representation of the socioeconomic influences on animal abandonments.

Another finding in this study is a positive association between the percentage of dog abandonments adopted and regional comprehensive income tax amounts, a proxy indicator of income level. This finding is consistent with a recent U.S. study that surveyed 6318 individuals across seven regions to examine how household income influences dog acquisition patterns [14]. That study found that individuals with lower incomes were more likely to acquire dogs through informal channels such as family or friends. In contrast, those with higher incomes were more inclined to adopt from shelters or purchase dogs, suggesting that income level influences the likelihood of adoption and the routes through which dogs are acquired. These findings combined imply that socioeconomic status significantly influences dog acquisition behaviors, highlighting the importance of considering income levels in animal welfare policies and shelter practices.

A recent Canadian study assessed social determinants as a predictor of animal relinquishment. The authors found that the four dimensions of the Canadian Index of Multiple Deprivation, including Ethnocultural Composition (e.g., the proportion of the population who self-identify as a visible minority), Situational Vulnerability (e.g., the proportion of the population that is low-income), Economic Dependency (e.g., the ratio of employment to population), and Residential Instability (e.g., the proportion of persons living alone), predicted increased risk of surrender across many shelter variables from 2016 to 2020 [2]. This Canadian study also proved the validity of using geospatial analysis to understand relationships between the status of human vulnerability and animal welfare, which led us to investigate if there is a spatial autocorrelation in the number of dog abandonments and percentage of dog abandonments adopted. Spatial dependence for the number of dog abandonments and the percentage of dog abandonments adopted was detected in our study, demonstrating the spatial relationship between the number of dog abandonments and predictors, including socioeconomic and veterinary cost indicators, which may support the use of spatial analysis to understand spatial relationships between the status of human vulnerability and animal welfare [2,15].

This study offers several methodological strengths that enhance the validity and applicability of its findings. First, we utilized publicly available nationwide datasets covering 162 regions across three consecutive years (2021–2023), providing a robust and comprehensive foundation for longitudinal analysis. Additionally, the use of objective veterinary service fee data—collected directly from more than 4000 veterinary clinics nationwide—adds a unique dimension to this research by capturing the real-world economic burdens experienced by pet guardians. Importantly, we applied spatial regression models, including Spatial Lag Models and Spatial Error Models, to account for spatial autocorrelation explicitly, a factor often overlooked in similar studies [1,8,10,16,17]. By correcting for spatial dependence, our analysis avoids biased estimations and reveals regional clustering patterns that are essential for informing targeted animal welfare interventions. The repeated validation of results across three years further reinforces the robustness of our conclusions and supports their relevance for policy planning and program development.

Despite its strengths, this study has several limitations that should be acknowledged. Although we made efforts to exclude unintentionally lost animals returned to their owners, the potential for underreporting or misclassification in the abandonment data remains, particularly due to regional variations in shelter intake practices and reporting accuracy. Furthermore, no significant spatial dependence was observed in the percentage of dog abandonments adopted for 2021 and 2022. This absence may be attributed to limited spatial heterogeneity in adoption behaviors or to the influence of shelter-specific policies and capacity, which were not accounted for in our model. Additionally, while the use of regional socioeconomic indicators such as income tax provided valuable macro-level insights, these variables may not fully capture individual or household-level decision-making processes regarding dog abandonment and the adoption of abandoned dogs. Lastly, this study focused solely on public animal shelters. It did not include data from private rescue organizations or informal rehoming efforts, which may follow different dynamics and could influence the overall patterns of abandonment and adoption.

Future research should build on these findings by incorporating a broader range of data sources and methodological approaches. Qualitative studies that explore pet guardians’ decision-making processes under economic or social stress would provide critical context to complement the quantitative patterns identified in this study. Expanding the analysis to more explicitly include cat abandonments and survival outcomes would enhance the comprehensiveness of species-specific insights. Furthermore, examining long-term outcomes for abandoned animals, such as euthanasia rates, transfers to other facilities, or the length of stay in shelters, would provide a more comprehensive picture of animal welfare beyond adoption alone. Ultimately, it would be beneficial to evaluate the impact of significant societal disruptions, such as the COVID-19 pandemic, on animal abandonment trends and the effectiveness of crisis-response policies in mitigating their effects on both human and animal well-being.

## 5. Conclusions

This study examined the factors influencing dog abandonment and adoption across 162 regions in the Republic of Korea from 2021 to 2023, utilizing socioeconomic indicators, veterinary service costs, and spatial regression models. Our findings reveal a consistent negative association between the number of people receiving unemployment benefits and the dog abandonment rates per 100,000 residents, contrasting with earlier Korean studies that did not account for population size. We also found that higher regional rabies vaccination costs were associated with lower abandonment rates, whereas other preventive care costs, such as DHPPL vaccination fees, were positively associated with abandonment.

Regarding adoption outcomes, comprehensive income tax amounts, a proxy for regional wealth, were positively associated with the percentage of abandoned dogs that were adopted, particularly in 2021 and 2023. These results align with international findings that show higher-income communities are more likely to adopt from shelters and have better access to adoption resources. Spatial analysis revealed strong spatial dependence in dog abandonment patterns across all three years, underscoring the importance of geographically targeted interventions. However, spatial dependence in adoption rates was not consistent and only emerged in 2023.

Our results suggest that targeted public support, such as subsidized veterinary services and community-based pet retention programs, may be convenient in economically vulnerable areas. Future research should incorporate qualitative data, consider cat-specific abandonment dynamics, and investigate the long-term effects of external shocks, such as the COVID-19 pandemic, on animal shelter outcomes.

## Figures and Tables

**Table 1:** Descriptive statistics of the variables used in the analysis for dog abandonments from 2021 to 2023.

Category	Variables	Observed Region	Mean	Standard Deviation	Lowest	Highest
Dependent variables	Number of dog abandonments per 100,000 residents in 2021	162	194.71	250.081	6.19	1624.73
	Number of dog abandonments per 100,000 residents in 2022	162	188.86	256.366	5.16	1398.81
	Number of dog abandonments per 100,000 residents in 2023	162	195.77	277.929	5.20	1592.81
	Percentage of dog abandonments adopted in 2021	162	0.45	0.183	0.09	0.94
	Percentage of dog abandonments adopted in 2022	162	0.39	0.168	0.04	0.80
	Percentage of dog abandonments adopted in 2023	162	0.38	0.187	0.04	0.83
Socioeconomic status	Number of people receiving unemployment benefits in 2021	162	10,926	8484	890	42,567
	Number of people receiving unemployment benefits in 2022	162	10,103	7832	895	39,853
	Number of people receiving unemployment benefits in 2023	162	10,361	8162	897	41,520
	Comprehensive income tax amounts (thousand KRW) in 2021	162	254,573,259	515,620,739	4500	4,599,753,000
	Comprehensive income tax amounts (thousand KRW) in 2022	162	291,234,481	537,801,113	8,113,000	4,865,729,000
	Comprehensive income tax amounts (thousand KRW) in 2023	162	309,935,284	573,151,575	9,326,000	5,209,045,000
	Local income per region (thousand KRW) in 2021	162	115,212,012	198,515,760	4,136,399	1,613,516,055
	Local income per region (thousand KRW) in 2022	162	143,140,693	248,078,132	4,422,652	1,996,875,421
	Local income per region (thousand KRW) in 2023	162	134,902,080	223,066,616	4,416,142	1,791,445,719
Veterinary costs ^1^	Initial examination fee amounts (thousand KRW)	162	9.09	1.570	5.00	17.50
	Re-visit examination fee amounts (thousand KRW)	162	7.07	1.630	4.50	15.00
	Consultation fee amounts (thousand KRW)	162	8.24	2.208	3.00	22.00
	Hospitalization fee amounts for small dogs (thousand KRW)	162	48.00	12.894	30.00	100.00
	DHPPL vaccination amounts (thousand KRW)	162	25.49	3.450	17.50	30.00
	Rabies vaccination amounts (thousand KRW)	162	24.10	3.837	15.00	30.00
	Kennel cough vaccination amounts (thousand KRW)	162	21.82	3.876	10.00	30.00
	Canine influenza vaccination amounts (thousand KRW)	162	34.83	3.819	25.00	40.00
	Complete blood count and reading fee amounts (thousand KRW)	162	33.54	4.489	25.00	60.00
	X-ray and reading fee amounts (thousand KRW)	162	39.78	4.907	20.00	52.50

^1^ Reported in 2024 by the Ministry of Agriculture, Food, and Rural Affairs (MAFRA), Republic of Korea; available at https://www.animalclinicfee.or.kr (accessed on 15 January 2025).

**Table 2:** Multiple regression model for number of dog abandonments per 100,000 residents in 2021.

Method	Category	Independent Variables	Coefficient	Standardized Coefficient	*p*-Value	Tolerance	VIF	Durbin–Watson	R^2^-Adjusted	F-Statistics	Significance Level
Enter	Socio- economic status	Number of people receiving unemployment benefits	−0.008	0.279	<0.001 ***	−3.777	1.362	1.477	0.357	7.864	<0.001 ***
		Comprehensive income tax amounts (thousand KRW)	2.251 × 10^−8^	−0.046	0.675	−0.42	3.054				
		Local income per region (thousand KRW)	−1.090 × 10^−7^	−0.086	0.436	−0.781	3.067				
	Veterinary costs	Initial examination fee amounts (thousand KRW)	6.273	0.039	0.652	0.452	1.904				
		Re-visit examination fee amounts (thousand KRW)	−10.613	−0.069	0.424	−0.801	1.867				
		Consultation fee amounts (thousand KRW)	9.932	0.088	0.215	1.246	1.24				
		Hospitalization fee amounts for small dogs (thousand KRW)	1.419	0.073	0.267	1.115	1.078				
		DHPPL vaccination amounts (thousand KRW)	26.632	0.367	0.003	2.999	3.755				
		Rabies vaccination amounts (thousand KRW)	−35.714	−0.548	<0.001 ***	−5.089	2.901				
		Kennel cough vaccination amounts (thousand KRW)	0.926	0.014	0.892	0.136	2.787				
		Canine influenza vaccination amounts (thousand KRW)	−13.919	−0.213	0.018	−2.39	1.979				
		Complete blood count and reading fee amounts (thousand KRW)	6.308	0.113	0.112	1.599	1.255				
		X-ray and reading fee amounts (thousand KRW)	3.386	0.066	0.318	1.002	1.1				
Enter	Socio- economic status	Number of people receiving unemployment benefits	−0.010	−0.343	<0.001 ***	−4.999	1.162	1.495	0.348	22.529	<0.001 ***
	Veterinary costs	DHPPL vaccination amounts (thousand KRW)	26.552	0.366	0.001 **	3.352	2.95				
		Rabies vaccination amounts (thousand KRW)	−34.522	−0.530	<0.001 ***	−5.217	2.547				
		Canine influenza vaccination amounts (thousand KRW)	−12.561	−0.192	0.020 *	−2.351	1.646				

* *p* < 0.05, ** *p* < 0.01, *** *p* < 0.001.

**Table 3:** Multiple regression model for number of dog abandonments per 100,000 residents in 2022.

Method	Category	Independent Variables	Coefficient	Standardized Coefficient	*p*-Value	Tolerance	VIF	Durbin–Watson	R^2^-Adjusted	F-Statistics	Significance Level
Enter	Socio- economic status	Number of people receiving unemployment benefits	−0.009	−0.270	<0.001 ***	−3.607	1.369	1.473	0.343	7.451	<0.001 ***
		Comprehensive income tax amounts (thousand KRW)	−1.505 × 10^−8^	−0.032	0.778	−0.283	3.048				
		Local income per region (thousand KRW)	−8.390 × 10^−8^	−0.081	0.468	−0.728	3.044				
	Veterinary costs	Initial examination fee amounts for dogs (thousand KRW)	8.085	0.050	0.575	0.562	1.903				
		Re-visit examination fee amounts (thousand KRW)	−16.731	−0.106	0.225	−1.218	1.868				
		Consultation fee amounts (thousand KRW)	7.634	0.066	0.357	0.924	1.24				
		Hospitalization fee amounts for small dogs (thousand KRW)	1.789	0.090	0.176	1.358	1.074				
		DHPPL vaccination amounts (thousand KRW)	15.062	0.203	0.104	1.637	3.753				
		Rabies vaccination amounts (thousand KRW)	−37.004	−0.554	<0.001 ***	−5.083	2.907				
		Kennel cough vaccination amounts (thousand KRW)	5.528	0.084	0.435	0.782	2.795				
		Canine influenza vaccination amounts (thousand KRW)	−7.818	−0.116	0.197	−1.296	1.978				
		Complete blood count and reading fee amounts (thousand KRW)	1.660	0.029	0.685	0.406	1.256				
		X-ray and reading fee amounts (thousand KRW)	5.739	0.11	0.103	1.639	1.100				
Enter	Socio- economic status	Number of people receiving unemployment benefits	−0.010	−0.311	<0.001 ***	−4.489	1.140	1.424	0.323	39.335	<0.001 ***
	Veterinary costs	Rabies vaccination amounts (thousand KRW)	−25.882	−0.387	<0.01 **	−5.593	1.140				

** *p* < 0.01, *** *p* < 0.001.

**Table 4:** Multiple regression model for number of dog abandonments per 100,000 residents in 2023.

Method	Category	Independent Variables	Coefficient	Standardized Coefficient	*p*-Value	Tolerance	VIF	Durbin–Watson	R^2^-Adjusted	F-Statistics	Significance Level
Enter	Socio- economic status	Number of people receiving unemployment benefits	−0.009	−0.259	<0.001 ***	−3.455	1.382	1.567	0.345	7.530	<0.001 ***
		Comprehensive income tax amounts (thousand KRW)	−1.446 × 10^−8^	−0.030	0.789	−0.268	3.031				
		Local income per region (thousand KRW)	−9.176 × 10^−8^	−0.074	0.510	−0.661	3.055				
	Veterinary costs	Initial examination fee amounts (thousand KRW)	17.538	0.099	0.262	1.127	1.902				
		Re-visit examination fee amounts (thousand KRW)	−23.497	−0.138	0.116	−1.581	1.867				
		Consultation fee amounts (thousand KRW)	7.921	0.063	0.377	0.886	1.24				
		Hospitalization fee amounts for small dogs (thousand KRW)	1.451	0.067	0.31	1.019	1.074				
		DHPPL vaccination amounts (thousand KRW)	17.024	0.211	0.089	1.71	3.753				
		Rabies vaccination amounts (thousand KRW)	−42.791	−0.591	<0.001 ***	−5.439	2.901				
		Kennel cough vaccination amounts (thousand KRW)	7.193	0.100	0.348	0.942	2.789				
		Canine influenza vaccination amounts (thousand KRW)	−8.568	−0.118	0.191	−1.312	1.98				
		Complete blood count and reading fee amounts (thousand KRW)	1.528	0.025	0.73	0.345	1.258				
		X-ray and reading fee amounts (thousand KRW)	6.267	0.111	0.1	1.654	1.1				
Enter	Socio- economic status	Number of people receiving unemployment benefits	−0.010	−0.293	<0.001 ***	−4.239	1.135	1.528	0.321	39.139	<0.001 ***
	Veterinary costs	Rabies vaccination amounts (thousand KRW)	−29.191	−0.403	<0.001 ***	−5.826	1.135				

*** *p* < 0.001.

**Table 5:** Multiple regression model for percentage of dog abandonments adopted in 2021.

Method	Category	Independent Variables	Coefficient	Standardized Coefficient	*p*-Value	Tolerance	VIF	Durbin–Watson	R^2^-Adjusted	F-Statistics	Significance Level
Enter	Socio- economic status	Number of people receiving unemployment benefits	2.157 × 10^−6^	0.100	0.240	1.18	1.362	1.591	0.156	3.296	<0.001 ***
		Comprehensive income tax amounts (thousand KRW)	1.356 × 10^−10^	0.381	0.003	3.012	3.054				
		Local income per region (thousand KRW)	−1.331 × 10^−10^	−0.144	0.258	−1.136	3.067				
	Veterinary costs	Initial examination fee amounts (thousand KRW)	−0.016	−0.136	0.175	−1.361	1.904				
		Re-visit examination fee amounts (thousand KRW)	−0.005	−0.047	0.635	−0.476	1.867				
		Consultation fee amounts (thousand KRW)	0.010	0.123	0.128	1.53	1.24				
		Hospitalization fee amounts for small dogs (thousand KRW)	0.001	0.071	0.347	0.944	1.078				
		DHPPL vaccination amounts (thousand KRW)	0.010	0.197	0.163	1.403	3.755				
		Rabies vaccination amounts (thousand KRW)	−0.000	−0.009	0.941	−0.075	2.901				
		Kennel cough vaccination amounts (thousand KRW)	−0.001	−0.025	0.836	−0.207	2.787				
		Canine influenza vaccination amounts (thousand KRW)	0.004	0.088	0.387	0.867	1.979				
		Complete blood count and reading fee amounts (thousand KRW)	0.002	0.058	0.478	0.711	1.255				
		X-ray and reading fee amounts (thousand KRW)	−0.001	−0.039	0.606	−0.517	1.100				
Enter	Socio- economic status	Comprehensive income tax amounts (thousand KRW)	1.220 × 10^−10^	0.343	<0.001 ***	4.615	1.000	1.464	0.112	21.299	<0.001 ***

*** *p* < 0.001.

**Table 6:** Multiple regression model for percentage of dog abandonments adopted in 2022.

Method	Category	Independent Variables	Coefficient	Standardized Coefficient	*p*-Value	Tolerance	VIF	Durbin–Watson	R^2^-Adjusted	F-Statistics	Significance Level
Enter	Socio- economic status	Number of people receiving unemployment benefits	1.711 × 10^−6^	0.080	0.372	0.895	1.369	1.588	0.064	1.849	0.041 *
		Comprehensive income tax amounts (thousand KRW)	4.006 × 10^−11^	0.128	0.337	0.964	3.048				
		Local income per region (thousand KRW)	−1.251 × 10^−11^	−0.018	0.890	−0.139	3.044				
	Veterinary costs	Initial examination fee amounts (thousand KRW)	0.003	0.032	0.764	0.301	1.903				
		Re-visit examination fee amounts (thousand KRW)	−0.013	−0.123	0.240	−1.181	1.868				
		Consultation fee amounts (thousand KRW)	0.011	0.139	0.104	1.636	1.240				
		Hospitalization fee amounts for small dogs (thousand KRW)	0.001	0.094	0.237	1.187	1.074				
		DHPPL vaccination amounts (thousand KRW)	0.002	0.034	0.818	0.231	3.753				
		Rabies vaccination amounts (thousand KRW)	0.006	0.148	0.258	1.136	2.907				
		Kennel cough vaccination amounts (thousand KRW)	−0.003	−0.065	0.614	−0.506	2.795				
		Canine influenza vaccination amounts (thousand KRW)	0.005	0.116	0.280	1.085	1.978				
		Complete blood count and reading fee amounts (thousand KRW)	0.002	0.066	0.438	0.778	1.256				
		X-ray and reading fee amounts (thousand KRW)	<0.001	−0.026	0.786	0.788	1.268				

* *p* < 0.05.

**Table 7:** Multiple regression model for percentage of dog abandonments adopted in 2023.

Method	Category	Independent Variables	Coefficient	Standardized Coefficient	*p*-Value	Tolerance	VIF	Durbin–Watson	R^2^-Adjusted	F-Statistics	Significance Level
Enter	Socio- economic status	Number of people receiving unemployment benefits	4.489 × 10^−7^	0.02	0.826	0.221	1.382	1.913	0.079	2.065	0.019
		Comprehensive income tax amounts (thousand KRW)	9.390 × 10^−11^	0.288	0.030 *	2.190	3.031				
		Local income per region (thousand KRW)	−1.577 × 10^−10^	−0.188	0.156	−1.426	3.055				
	Veterinary costs	Initial examination fee amounts (thousand KRW)	0.006	0.049	0.64	0.469	1.902				
		Re-visit examination fee amounts (thousand KRW)	−0.014	−0.122	0.239	−1.183	1.867				
		Consultation fee amounts (thousand KRW)	0.008	0.098	0.247	1.162	1.24				
		Hospitalization fee amounts for small dogs (thousand KRW)	0.001	0.087	0.267	1.113	1.074				
		DHPPL vaccination amounts (thousand KRW)	0.002	0.033	0.822	0.225	3.753				
		Rabies vaccination amounts (thousand KRW)	0.007	0.148	0.253	1.148	2.901				
		Kennel cough vaccination amounts (thousand KRW)	−0.005	−0.113	0.371	−0.897	2.789				
		Canine influenza vaccination amounts (thousand KRW)	0.009	0.181	0.090	1.705	1.98				
		Complete blood count and reading fee amounts (thousand KRW)	0.001	0.016	0.850	0.19	1.258				
		X-ray and reading fee amounts (thousand KRW)	0.004	0.103	0.198	1.292	1.1				
Enter	Socio- economic status	Comprehensive income tax amounts (thousand KRW)	6.886 × 10^−11^	0.211	0.006 **	2.737	1.000	1.794	0.039	7.490	0.007 **

* *p* < 0.05, ** *p* < 0.01.

**Table 8:** Results of Spatial Lag Model (SLM) assessing the number of dog abandonments per 100,000 residents and spatial dependence from 2021 to 2023.

Variable		2021		2022		2023	
		Coefficient	*p*-Value	Coefficient	*p*-Value	Coefficient	*p*-Value
Constant		−0.431	0.978	1.540	0.927	1.359	0.940
Socioeconomic status	Number of people receiving unemployment benefits	−0.008	<0.001 ***	−0.009	<0.001 ***	−0.009	<0.001 ***
	Re-visit examination fee amounts (thousand KRW)	8.287	0.422	−21.930	0.037 *	−28.869	0.011 *
Veterinary costs	Consultation fee amounts (thousand KRW)	14.647	0.013 *	13.216	0.034 *	14.249	0.034 *
	Hospitalization fee amounts for small dogs (thousand KRW)	1.821	0.049 *	2.247	0.021 *	1.958	0.062
	DHPPL vaccination amounts (thousand KRW)	27.647	<0.001 ***	16.672	0.016 *	18.681	0.012 *
	Rabies vaccination amounts for dogs (thousand KRW)	−32.551	<0.001 ***	−34.494	<0.001 ***	−39.514	<0.001 ***
	Canine influenza vaccination amounts (thousand KRW)	−8.630	0.032 *	−1.656	0.698	−2.019	0.660
	Complete blood count and reading fee amounts (thousand KRW)	8.983	0.001 **	4.685	0.117	4.596	0.153
	X-ray and reading fee amounts (thousand KRW)	5.23692	0.019 **	8.751	<0.001 ***	9.632	<0.001 ***
Rho (ϱ)		0.213	<0.001 ***	0.185	<0.001 ***	0.202	<0.001 ***
R^2^		0.543	-	0.505	-	0.506	-
Log likelihood		−1608.930	-	−1622.210	-	−1640.770	-
Akaike info criterion		3247.850	-	3274.420	-	3311.540	-
Schwarz criterion		3300.670	-	3327.240	-	3364.360	-
Likelihood Ratio test		18.480	<0.001 ***	12.266	<0.001 ***	14.556	<0.001 ***
Breusch–Pagan test		416.265	<0.001 ***	296.500	<0.001 ***	309.063	<0.001 ***

* *p* < 0.05, ** *p* < 0.01, *** *p* < 0.001.

**Table 9:** Results of ordinary least squares model assessing the percentage of dog abandonments adopted and spatial dependence from 2021 to 2023.

Variable		2021		2022		2023	
		Coefficient	*p*-Value	Coefficient	*p*-Value	Coefficient	*p*-Value
Constant		<0.001	0.973	−0.001	0.913	−0.003	0.859
Socioeconomic status	Comprehensive income tax amounts (thousand KRW)	<0.001	<0.001 ***	<0.001	0.173	<0.001	0.003 **
Veterinary costs	Consultation fee amounts (thousand KRW)	0.011	0.045 *	0.010	0.056	0.007	0.231
Lambda (λ)		-	-	-	-	-	-
R^2^		0.749	-	0.702	-	0.655	-
Log likelihood		155.908	-	164.420	-	138.911	-
Akaike info criterion		−283.816	-	−300.840	-	−249.823	-
Schwarz criterion		−234.515	-	−251.540	-	−200.522	-
Likelihood Ratio test		-	-	-	-	-	-
Breusch–Pagan test		85.289	<0.001 ***	89.882	<0.001 ***	90.402	<0.001 ***
Moran’s I (error)		−0.061	0.381	0.028	0.528	72.491	<0.001 ***
Lagrange Multiplier (lag)		0.001	0.982	0.242	0.623	0.124	0.023
Robust LM (lag)		0.566	0.452	0.060	0.807	3.465	0.063
Lagrange Multiplier (error)		1.051	0.305	0.219	0.640	0.320	0.571
Robust LM (error)		1.616	0.204	0.038	0.847	4.386	0.03624 *

* *p* < 0.05, ** *p* < 0.01, *** *p* < 0.001.

## Data Availability

The original contributions presented in this study are included in the article/Supplementary Materials. Further inquiries can be directed to the corresponding authors.

## Supplementary Materials

The following supporting information can be downloaded at: https://www.mdpi.com/article/10.3390/ani15111613/s1. Table S1: The Numbers of Dog Abandonments and Their Survival by Region from 2021 to 2023 used in the Analysis; Table S2: Results of Ordinary Least Square (OLS) Model, Spatial Lag Model (SLM), and Spatial Error Model (SEM) Assessing the Number of Dog Abandonments per 100,000 residents and Spatial Dependence for 2021; Table S3: Results of Ordinary Least Square (OLS) Model, Spatial Lag Model (SLM), and Spatial Error Model (SEM) Assessing the Number of Dog Abandonments per 100,000 residents and Spatial Dependence for 2022; Table S4: Results of Ordinary Least Square (OLS) Model, Spatial Lag Model (SLM), and Spatial Error Model (SEM) Assessing the Number of Dog Abandonments per 100,000 residents and Spatial Dependence for 2023; Table S5: Results of Ordinary Least Square (OLS) Model, Spatial Lag Model (SLM), and Spatial Error Model (SEM) Assessing the Percentage of Dog Abandonments Adopted and Spatial Dependence for 2023; Figure S1: Spatial Distribution of the Number of Dog Abandonments Per 100,000 Residents in 2021 (a), 2022 (b), and 2023 (c); Figure S2. Spatial Distribution of the Percentage of Dog Abandonments Adopted Residents in 2021 (a), 2022 (b), and 2023 (c).
